# Surface Characteristics and Artificial Weathering Resistance of Oil-Based Coatings on the Chemically and Thermally Modified Short-Rotation Teak Wood

**DOI:** 10.3390/ma17153881

**Published:** 2024-08-05

**Authors:** Resa Martha, Béatrice George, Christine Gérardin-Charbonnier, Emmanuel Fredon, Istie S. Rahayu, Wayan Darmawan, Philippe Gérardin

**Affiliations:** 1 Faculty of Science and Technologies, Université de Lorraine, INRAE, LERMAB, 54000 Nancy, France; resa.martha@univ-lorraine.fr (R.M.); beatrice.george@univ-lorraine.fr (B.G.); christine.gerardin@univ-lorraine.fr (C.G.-C.); emmanuel.fredon@univ-lorraine.fr (E.F.); 2 Department of Forest Products, Faculty of Forestry and Environment, IPB University, Bogor 16680, Indonesia; istiesr@apps.ipb.ac.id

**Keywords:** accelerated ageing, chemical and thermal modification, linseed oil, hydrophobic film, fast growing teak wood, surface property, tung oil, wettability

## Abstract

Improving the durability of short-rotation wood can be achieved through chemical and thermal modification. Chemical and thermal modification can have an impact on the physicochemical properties of wood, which can affect wood’s surface characteristics and its resistance to weathering. The purpose of this study was to investigate the surface characteristics and artificial weathering resistance of chemically and thermally modified short-rotation teak wood coated with linseed oil (LO)-, tung oil (TO)-, and commercial oil-based coatings consisting of a mixture of linseed oil and tung oil (LT) and commercial oil-based polyurethane resin (LB) coatings. The short-rotation teak woods were prepared in untreated and treated with furfuryl alcohol (FA), thermal treatment (HT) at 150 and 220 °C, and combination of glycerol–maleic anhydride (GMA) impregnation with thermal treatment at 150 and 220 °C. The surface characteristics measured were surface free energy, wettability, Persoz hardness, bonding quality, and color changes before and after artificial weathering exposure. The results showed that chemical and thermal modifications treatment tended to reduce total surface free energy (SFE), hardness, wettability, and bonding quality. FA and GMA at 220 °C treatments provided homogenization effect on surface characteristics, especially in total SFE and wettability. The total SFE of untreated wood ranged from 45.00 to 51.13 mN/m, and treated wood ranged from 40.58 to 50.79 mN/m. The wettability of oil-based coating according to K-value ranged from 0.20 to 0.54. TO presented better photostability than LO. Short-rotation teak wood coated with oil-based commercial coatings presented better weathering resistance compared to pure natural drying oil. Commercial oil-based coatings provided better weathering protection for the chemically and thermally modified teak wood. The application of oil-based coatings on chemically and thermally modified short-rotation teak is being considered for the development of a better wood-protection system.

## 1. Introduction

Wood is a raw material that is widely used in the furniture industry. Long-rotation teak wood (*Tectona grandis* Linn. F) is one of the most valuable hardwood species which is widely used for furniture and exterior applications in large quantities. Long-rotation teak wood has outstanding properties, such as high dimensional stability, high durability, an aesthetic wood color and pattern, good workability, and high economic value [1,2,3,4,5]. However, long-rotation teak has a long cutting cycle (40 to 60 years), which can cause limited availability in the market. Short-rotation teak is currently being developed, especially in Indonesia, to fulfill the increasing demand for long-rotation teak. Short-rotation teak wood has a short cutting cycle (7 to 15 years) but is also low quality, especially in regard to its dimensional stability and durability [5,6,7,8]. Previous studies have enhanced the quality of short-rotation teak wood through chemical and thermal modification methods [9,10,11,12,13]. Moreover, the technological properties of sapwood and heartwood of short-rotation teak, such as decay resistance, dimensional stability, or color, can be homogenized after chemical and thermal modification [14].

Wood used for exterior applications that has been exposed to the ground and various different environmental factors is subjected to different degradations. The combination of light, heat, and water can cause the depolymerization of lignin and cellulose when wood products are exposed to outdoor exposures [15,16]. The effects of changes in chemical components due to outdoor exposure cause discoloration, roughening, and cracking [17,18]. Our previous studies reported that chemical and thermal modifications, such as thermal modification [9], furfurylation [10], and glycerol–maleic anhydride (GMA) [11], have the potential to improve the dimensional stability and durability of short-rotation teak wood, leading to its use for exterior applications. In order to enhance the durability and service life of exterior applications, surface coatings can be applied to modified wood surfaces [19,20,21]. Chemical and thermal modifications can alter the hydrophilic properties of wood, thus impacting the coating adhesion and other wood surface properties. Our previous study found that chemically and thermally treated short-rotation teak wood is not suitable for water-based coatings due to a decrease in free hydroxyl groups leading to the reduced hygroscopicity of the wood [22], even if the application of water-based wood coatings can be a solution to reduce environmental issues. Meanwhile, no studies have been performed on improving the wood quality of conventional teak wood through chemical or thermal modifications due to its high natural durability and good dimensional stability.

The utilization of renewable natural oils as raw materials for coating wood surfaces, such as linseed oil and tung oil, appears as an attractive alternative in the paint and coatings industry [23,24]. Linseed oil and tung oil are the most representative drying oils for finishing, which are relatively inexpensive and environmentally friendly. Linseed oil, extracted from the seeds of flax (*Linum usitatissimum*), is the most widely utilized natural oil for wood applications in Europe. Tung oil, obtained by pressing seeds from the nuts of the Tung tree (*Vernica fordii*), is widely used as an alternative to linseed oil in the American and Asian markets. Linseed oil and tung oil consist of triglycerides with unsaturated fatty acids [25]. Linseed oil consists of 53% linolenic acid containing three unconjugated double bonds, while tung oil consists of more than 82% alpha-eleostearic acid containing three conjugated double bonds [26]. Oils which are enriched with unsaturated fatty acids, such as α- or γ-linolenic acid or eleostearic acid, have application as wood varnishes [27]. These oils have outstanding properties in terms of surface-finishing durability due to their chemical composition and film-formation mechanism. The high content of unsaturated fatty acids in drying oils enables a rapid reaction with atmospheric oxygen, resulting in autoxidation and polymerization. This process leads to the crosslinking of unsaturated fatty acids, which hardens the oil into a solid film [28].

The quality of the wood product manufactured depends on the quality of the final coating to the wood product. The durability of the coating film is determined by the bonding quality between the coating material and the wood surface [22,29,30]. The interaction between the wood surface and the coating material can be assessed through the observation of thermodynamic wetting parameters, such as contact angle, surface free energy (SFE), and wettability [30,31]. The surface characteristics of short-rotation teak wood modified by chemical and thermal modification treatments still require further investigation; therefore, it is the focus of this research. The synergy of wood modification and coating applications needs to be achieved to expand the opportunities for the utilization of short-rotation teak wood that provides good resistance against weathering. The purpose of this study was to investigate the surface characteristics and artificial weathering resistance of chemically and thermally modified short-rotation teak wood coated with linseed oil-, tung oil-, and oil-based commercial coatings. In addition, it was possible to evaluate the homogenization of wood surface characteristics in sapwood and heartwood after chemical and thermal modification.

## 2. Materials and Methods

### 2.1. Sample Preparation

Short-rotation teak trees were sourced from a plantation forest in Bogor, West Java, Indonesia (6°33′15″ S/106°40′07″ E). These trees were grown under conditions featuring an average annual rainfall of 2000–3000 mm and temperatures ranging between 15 and 31 °C. The 13-year-old teak trees had an average diameter of 27 cm at breast height. Wood blocks designated for chemical and thermal modifications were cut to dimensions of 200 × 50 × 20 mm^3^ (L × R × T). These samples were air-dried to achieve a final moisture content of 12–15% under controlled conditions of a 23 ± 2 °C temperature and 50 ± 5% relative humidity. Samples of wood blocks were collected from sapwood (SW), transition wood (TW) containing an equal proportion of sapwood and heartwood, and heartwood (HW). The research included an investigation into six treatment methods: untreated, furfurylation, thermal treatment at 150 °C, thermal treatment at 220 °C, GMA–thermal treatment at 150 °C, and GMA–thermal treatment at 220 °C. Following the treatment process, the wood species were cut into smaller pieces for further surface characterization. TW part was divided into transition sapwood (TS) and transition heartwood (TH).

### 2.2. Chemical and Thermal Modification

Chemical and thermal modification were performed according to Martha et al. [14]. Chemical modification was carried out by impregnating a solution of 45% furfuryl alcohol and 5% tartaric acid in aqueous solution. Impregnation was performed by the vacuum-pressure method in an autoclave. The vacuum procedure was conducted under vacuum conditions of 8–10 kPa for 15 min, involving immersion in FA solution under vacuum conditions of 4–5 kPa for another 15 min. Subsequently, the pressure treatment was applied at a pressure of 1200 kPa for 30 min. The polymerization stage was conducted at 120 °C for 16 h under a nitrogen atmosphere.

Thermal modification was conducted at 150 and 220 °C under nitrogen conditions. The oven temperature was elevated at a heating rate of 20 °C per minute from room temperature to the designated target temperature, and then it was maintained for a duration of 20 h.

Combination of chemical treatment using glycerol–maleic anhydride and thermal treatment was conducted by impregnation with 10% w/w aqueous solution of GMA, followed by thermal treatment at 150 and 220 °C under inert conditions. GMA solution was made by reacting 1 mol of glycerol (92.09 g mol^−1^) and 2 mol of maleic anhydride (98.06 g mol^−1^), followed by heating at 80 °C for 3 h. The impregnation phase involved a vacuum treatment at 8–10 kPa for 30 min, followed by a pressure treatment at 1200 kPa for 60 min. Curing temperatures of 150 and 220 °C were then applied for a duration of 20 h under nitrogen conditions.

### 2.3. Surface Free-Energy Measurement by Tensiometer

The determination of surface free energy (SFE) was performed by measuring dynamic contact angles using the Force Tensiometer—K100 (Krüss GmbH, Hamburg, Germany), a multi-flexible instrument for analyzing surfaces and interfaces. In order to determine the SFE, the dynamic contact angle was measured using three reference liquids where the surface tension of polar and dispersion fractions was known (Table 1). Dynamic contact angle was measured by the Wilhelmy plate method. A wood plate of size 10 × 10 × 3 mm^3^ (R × T × L) was prepared and then sanded to obtain the same level of surface roughness. Three measurements for each liquid were performed on a wood plate. The measurement was carried out at a room temperature of 25 °C. The wood plate was immersed and pulled out along its radial direction into the liquid to determine the advancing and receding contact angles. The SFE of a wood plate was calculated using the advancing contact angles of three liquids. The calculation of SFE was performed using ADVANCE software version 1.14 (Krüss GmbH, Hamburg, Germany) based on the dynamic contact angle of each liquid.

Numerous methods have been employed to calculate the SFE of wood. In this study, Owens, Wendt, Rabel, and Kaelble (OWRK) method [32] was used to calculate the SFE of untreated and treated short-rotation teak wood. The basic method in calculating the SFE for a solid ($\gamma_{S}$) using the contact angle value (θ) is based on Young’s equation [33], as follows:

(1) $$\gamma_{S}=\gamma_{SL}+\gamma_{L}\cos\theta$$

where $\gamma_{S}$ is the surface tension of solid, $\gamma_{SL}$ is the interfacial tension between liquid and solid, $\gamma_{L}$ is the surface tension of liquid, and θ is the contact angle between a solid and liquid. If the values of $\gamma_{L}$ and θ are known, it is not possible to determine the SFE directly from Equation (1) because there are still two unknown variables, $\gamma_{S}$ and $\gamma_{SL}$.

According to the Fowkes method, the interfacial tension, $\gamma_{SL}$, is calculated based on the two surface tensions, $\gamma_{S}$ and $\gamma_{L}$, and the similar interactions between the phases. In this approach, these interactions are interpreted as the geometric mean of a disperse component ($\gamma^{d}$) and a polar component ($\gamma^{p}$) of the surface tension or surface free energy:

(2) $$\gamma_{SL}=\gamma_{S}+\gamma_{L}-2\left(\sqrt{\gamma_{S}^{d}\gamma_{L}^{d}}+\sqrt{\gamma_{S}^{p}\gamma_{L}^{p}}\right)$$

Substituting Equation (2) with Young’s Equation (1) generates the following work equation, Equation (3):

(3) $$\gamma_{L}\left(1+\cos\theta \right)=2\sqrt{\gamma_{S}^{d}\gamma_{L}^{d}}+2\sqrt{\gamma_{S}^{p}\gamma_{L}^{p}}$$

### 2.4. Contact Angle Measurement

The dynamic contact angle was measured according to Engonga et al. [34]. It was evaluated by the sessile drop method, using the DSA10 MK2 Drop Shape Analysis system (Krüss GmbH, Hamburg, Germany). Wood samples with a size of 50 × 50 × 10 mm^3^ were evaluated at SW, HW, TS, and TH for untreated and treated wood. Three drops per sample were measured for each oil-based coating. The dynamic contact angle was automatically recorded every 3 s up to 60 s. The contact angle of each droplet on the wood surface was measured both on the left and right side of the droplet, and then the mean values were calculated automatically. A total of 21 data points were collected for each droplet to generate a curve of contact angle versus wetting time.

### 2.5. Determination of Contact-Angle Change Rate (Wettability)

The equilibrium contact angle was determined using a segmented regression model that was identified through the transition point between contact angle and time, using the PROC NLIN procedure in SAS (SAS STAT 9.1, SAS Institute Inc., Cary, NC, USA). Furthermore, the contact-angle change rate (K-value) in the S/G model [35] was used to evaluate wettability quantitatively. The S/G model equation is expressed in Equation (4) as follows:

(4) $$\theta =\frac{\theta_{i}\times \theta_{e}}{\theta_{i}+\left(\theta_{e}-\theta_{i}\right)exp\left[K\left(\frac{\theta_{e}}{\theta_{e}-\theta_{i}}\right)t\right]}$$

where θ is the contact angle at a certain time, $\theta_{i}$ is the initial contact angle, $\theta_{e}$ is equilibrium contact angle, K is the constant contact angle change rate, and t is wetting time. A non-linear regression model was used to calculate the K-value, using the defined function to fit S/G equation by XLSTAT (XLSTAT Addinsoft, Denver, CO, USA).

### 2.6. Coating Application

The oil-based coatings used in this study were a linseed oil coating, tung oil coating, commercial oil-based coatings with a mixture of linseed oil and tung oil provided by “Le Tonkinois”, and commercial oil-based polyurethane resin coatings from “Libéron” (Table 2). In order to accelerate the drying time, the initial layers for linseed oil and tung oil coatings were added with Co, Zi, and Ca driers. In this study, we used industrial driers, namely CoZi 69 complex from Octa soligen or Valirex (6% Co and 9% Zi) mixed with Ca. The viscosity of oil-based coatings was measured using Fungilab V210003 Smart Series L Rotational Viscometer (FUNGILAB Inc., New York, NY, USA). The solids content (Sc) was determined by the gravimetric method. The fundamental properties of the oil-based coatings are presented in Table 2.

Table 3 shows the different coating treatments applied on wood surfaces for each layer. This mixture was applied to the first and second layers. The commercial coatings (LT and PU) were applied according to the instructions for use from the manufacturer. Wood samples with a size of 75 × 50 × 10 mm^3^ were prepared from SW, TW, and HW of untreated and treated short-rotation teak wood. The wood samples were sanded parallel to the longitudinal direction, using abrasive paper of 180 grit. All coatings were manually applied in three layers by brush. The spreading rate was 50 g/m^2^ for the first and second layers, and 25 g/m^2^ for the third layer. The first and second layers were dried for 24 h. The third layer was applied after the wood surface was sanded by abrasive paper of 400 grit. After the coating application, wood samples were dried for a week before being used for surface characterization.

### 2.7. Hardness by Persoz Pendulum

The hardness of coating layers was assessed for oil-based coating materials applied to untreated and treated short-rotation teak wood. It was evaluated by a Persoz pendulum according to EN ISO 1522 [36]. Untreated and treated wood samples from different oil-based coatings were prepared with dimensions of 75 × 50 × 10 mm^3^. The sample was placed on a horizontal panel, and a pendulum with 8 mm diameter tungsten–carbide balls at 50 mm apart was placed on it. The pendulum was deflected through an angle of 12°, without lateral displacement of the fulcrum. The pendulum was released and simultaneously started the stopwatch. The damping time from 12° to 4° displacement was recorded and represented the Persoz hardness of the tested surface. Three measurements per each sample were recorded.

### 2.8. Cross-Cut Test

Untreated and treated wood pieces in the size of 75 × 50 × 10 mm^3^ were prepared from different coatings. The cross-cut test was performed on the coated wood surfaces for the SW, TW, and HW parts. The coating film on each specimen was scraped off with a uniform pressure of about 45° to the grain direction. We removed the loose paint from the cutting area. Two measurements were performed on each sample surface. Bonding quality was classified according to the ISO 2409:2020 standard [37] (Table 4).

### 2.9. Artificial Weathering Test

The untreated and treated wood with different oil-based coatings were prepared from the SW, HW, and TW parts. The prepared wood samples of 75 × 50 × 10 mm^3^ were exposed to accelerated weathering test using a QUV Accelerated Weathering Tester (Q-Lab, Westlake, OH, USA). The coated woods were exposed by the cycle described in Table 5. The weathering cycle began with a condensation phase for 24 h at 45 °C. The condensation phase was followed by UV exposure for 2.5 h and water spray for 30 min, which were repeated 48 times. The UV irradiation level was 0.89 W/m^2^ at UVA-340 nm source type. The spray nozzle size was 200 m, with a flow rate of 6–7 L/min. The total duration of one cycle was 168 h (one week). The exposure was interrupted every week, and wood samples were taken out for color evaluation. The artificial weathering test was performed for 2016 h (12 weeks).

### 2.10. Color Changes

Color measurements were performed on the CIE L*, a*, and b* system using an X-Rite spectrophotometer SP60 (X-Rite Pantone, New York, NY, USA). Samples of 75 × 50 × 10 mm^3^ were analyzed in SW, HW, TS, and TH for each treatment. Color measurements were recorded before the accelerated weathering test and every week during the accelerated weathering test. The parameters L*, a*, and b* for each specimen were measured at three contiguous locations on the wood surface. Total color change (∆*E*) was calculated based on CIELAB, according to the following formula (Equation (5)):

(5) $$∆E=\sqrt{{\left(∆L\right)}^{2}+{\left(∆a\right)}^{2}+{\left(∆b\right)}^{2}}\;$$

where L* is the lightness; a* and b* describe the chromatic coordinates on the green-red color and blue-yellow axes, respectively; ΔL*, Δa*, and Δb* are the differences between before and after accelerated weathering test; and ∆E is total color changes.

### 2.11. Data Analysis

One-way analysis of variance (ANOVA) for a completely randomized design with two factors (wood part and treatment) was carried out to characterize the values of contact angle of reference liquids, SFE components, K-value of oil-based coatings, and hardness. The mean differences of the values between the treatments were tested using Duncan’s multiple range test at a 5% significant level.

## 3. Results and Discussions

### 3.1. Contact Angle and Surface Free-Energy Components

The results in Table 6 show contact angles obtained with the reference liquids for untreated and treated in different parts of short-rotation teak wood. The contact angle of glycerol as a polar liquid was not significantly different either between treatments or between wood parts. Meanwhile, the water contact angle increased significantly after treatment, which indicated that chemical and thermal modification could decrease the wettability of water on the wood surface. The high contact angle of water might be caused by the decrease in hydrophilic properties of short-rotation teak wood after chemical and thermal modification treatment. Chemical and thermal modifications reduced the free hydroxyl group, indicating that chemically and thermally modified short-rotation teak woods become more hydrophobic [10,11,14]. The contact angle of water in heartwood was higher than that of sapwood. A similar phenomenon was observed for transition wood in which transition sapwood had a lower contact angle than transition heartwood. This result is explained by the presence of high extractive content in the heartwood resulting in more hydrophobic properties [8]. Diiodomethane is a polar liquid with low surface tension, leading to a smaller contact angle than glycerol and water. The contact angle of diiodomethane did not change significantly in wood treated with HT150 and GMA150, while it tended to increase in wood treated with FA, HT220, and GMA220. This result could give an indication that the wettability of short-rotation teak wood might decrease after FA, HT220, and GMA220 treatments. Similar to the trend of the contact angle of water, heartwood and transition heartwood presented a higher contact angle of diiodomethane compared to sapwood and transition sapwood. The results of contact angle observed with different liquids were used to calculate the surface free energy of untreated and treated in different parts of short-rotation teak wood.

The dispersive component, polar component, and total SFE were calculated using the OWRK method, with the reference liquids, as presented in Table 7. The dominant component of the surface free energy was the dispersive component. This is characteristic of the main wood-component polymers [38]. The higher dispersive component can be explained by the higher interaction ability of the dispersive parts within cellulose, particularly from the available carbon–oxygen and carbon–carbon bonds [38,39]. The polar component was higher in heartwood than sapwood due to the higher extractive content in heartwood. A higher extractive content might introduce more polar functional groups (e.g., hydroxyl, carboxyl, and methoxy groups) to the wood surface, therefore increasing the polar component. The total SFE of untreated short-rotation teak wood in different wood parts ranged from 45.00 to 51.13 mN/m, and it decreased after chemical and thermal modification. Furfurylated wood presented the lowest surface free energy. Meanwhile, the SFE of wood treated by thermal and GMA treatments decreased as the heating temperature increased. Chemical and thermal modifications reduce the hygroscopicity of wood due to chemical changes which increase the hydrophobicity of wood after modification [10,11,40]. The increase in hydrophobicity can lead to changes in SFE components [41]. The result in Table 7 shows no significant difference between transition sapwood and transition heartwood according to Duncan’s multiple range test. This result gave an indication that transition sapwood and transition heartwood were homogeneous in total SFE after FA and GMA220 treatments. Martha et al. [30] reported that the SFE value can be used to quantitatively determine the wettability in term of K-value. The high SFE of the wood causes the high energy on the wood surface, which can allow the liquid to spread and penetrate the surface faster [29,30,42].

### 3.2. Wettability

The wettability of an oil-based coating on various wood surfaces can be quantitatively assessed using an S/G model. The K-value was used as the parameter for quantifying the wettability of the oil-based coating on the surface of the untreated and treated short-rotation teak wood. A high K-value indicates that the liquid spreads and penetrates the wood surface faster [35]. Table 8 presents different wood parts for untreated and treated of short-rotation teak wood. In general, the K-value of the oil-based coatings decreased after chemical and thermal modification treatments. This result indicated that chemical and thermal modification decreased the wettability of short-rotation teak wood. This result was attributed to the total SFE values that decreased after chemical and thermal modification treatments. The results in Table 8 also show that the FA treatment has the lowest K-value, which is similar to the phenomenon observed in the total SFE value. Moreover, the K-value decreased as the heating temperature increased in both thermal and GMA treatments. Previous studies reported that a decrease in the total SFE value contributes to the decrease in wettability of coating material on wood surface [22,29,30]. A lower K-value was generally observed in heartwood part compared to sapwood. The high extractive content of heartwood influences the surface chemistry of wood by masking or occupying some of the oxygen sites present in wood components, and therefore it affects the wettability and surface energy characteristics [43,44]. The K-value of oil-based coatings between transition sapwood and transition heartwood did not differ significantly according to Duncan’s multiple range test for wood treated by FA and GMA220 treatments. The results indicate that short-rotation teak board consisting of sapwood and heartwood was homogenized in wettability using the oil-based coating after FA and GMA220 treatments.

As shown in Table 8, the natural drying oil (linseed oil and tung oil) presented a higher K-value compared to the commercial oil-based coating. This might be due to the differences in viscosity of the oil-based coating affecting wettability. According to Table 2, the natural drying oil exhibited a lower viscosity than commercial oil-based coating. This result indicates that the natural drying oil with lower viscosity provided better wettability on untreated and treated short-rotation teak wood. Wettability decreases with high viscosity because the greater cohesion force between the liquid molecules makes it more difficult to spread on the wood surfaces [45,46]. As compared to linseed oil and tung oil, tung oil had a higher viscosity than linseed oil. However, the K-values between linseed oil and tung oil were almost similar, thus indicating that tung oil performed better in regard to penetration and spreading on the wood surface than linseed oil. Our previous work investigated the surface characteristics of water-based coatings on chemically and thermally modified short-rotation teak [22]. The results reported that K-values range from 0.05 to 0.14 for water-based coatings. In this study, the K-value resulting from the oil-based coating on the surface of short-rotation teak wood was much higher (0.20–0.54) compared to that of the water-based coating. This indicates that the wettability of the oil-based coating on chemically and thermally modified short-rotation teak wood was much better compared to the water-based coating.

### 3.3. Persoz Hardness

**Figure 1:**
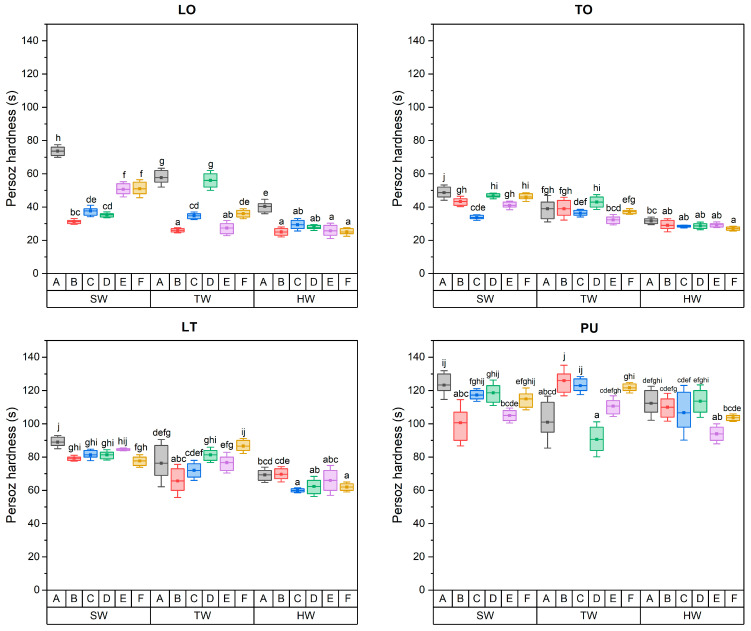
Persoz hardness of untreated and treated short-rotation teak wood for different oil-based coatings. Note: A = untreated, B = furfurylation, C = thermal treatment at 150 °C, D = thermal treatment at 220 °C, E = C = GMA–thermal treatment at 150 °C and F = C = GMA–thermal treatment at 220 °C. The a–j values followed by the same do not differ significantly (α = 0.05) based on the Duncan test.

The influence of the various oil-based coatings on the Person hardness is presented in Figure 1. The Persoz hardness of oil-based coating on short-rotation teak wood decreased due to chemical and thermal modification. This might be due to the chemical and thermal modification reducing the number of reactive hydroxyl groups on the wood surface which are important for bonding with the oil-based coating. A reduction in reactive hydroxyl groups could produce weaker bonds, leading to a decrease in hardness. Among the wood parts, the Person hardness of the oil coating material in sapwood showed a high value, followed by the transition and heartwood. The presence of extractives on the surface of heartwood might generate a less cohesive coating and therefore reduce the Persoz hardness of oil-based coatings. The antioxidant properties of phenolic extractives present in wood might influence the drying process of oils and potentially reduce the hardness. Indeed, radical scavenging properties of phenolic extractives are known to slow down the hardening process of siccative oil involving radical polymerization of unsaturated moieties of fatty acid alkyl chains.

The Persoz hardness of oil-based coatings for untreated and treated short-rotation teak wood was in the range of 23–76 s for LO, 26–52 s for TO, 58–92 s for LT, and 84–140 s for PU. The Persoz hardness of natural drying oil was lower than commercial oil-based coating. The main components in linseed oil include linolenic and linoleic acids, and tung oil is mainly composed of eleostearic acid [25,26,27]. Meanwhile, commercial coating materials contain synthetic polyurethane resins, which have a more complex chemical structure. This might be the reason for the harder film produced. The application of natural drying oils on wood surfaces is restricted by time-consuming polymerization [47]. The drying process of natural oils was slower compared to commercial oil-based coating. The curing process might involve exposure to oxygen, leading to less consistent film formation. The hardness of oil-based coatings could possibly affect the bonding quality of short-rotation teak.

### 3.4. Bonding Quality

The boding qualities of untreated and treated short-rotation teak wood with different oil-based coatings are presented in Table 9. The bonding quality of untreated short-rotation teak was slightly better than that of treated teak. Chemical and thermal modification could modify the chemical composition of the wood surface, and this might reduce its polarity and increase its hydrophobicity. These changes could affect how well oil-based coatings adhere to and dry on the surface. Furthermore, the decrease in SFE and K-value led to a decrease in the bonding quality of chemically and thermally modified short-rotation teak. Previous studies reported that bonding quality on the surface of short-rotation teak wood decreases due to due to the lower SFE value and wettability after chemical and thermal modification treatment [12,22].

The bonding quality of TO coating was slightly better than LO coating. Tung oil had a faster drying and polymerization time; therefore, the final coating formed was harder and produced better adhesion to the wood surface. Tung oil has a higher reactivity than the non-conjugated double bonds of linolenic acid in linseed oil, which results in a more effective reaction and higher cross-linking density in the oxidation process [48,49]. As shown in Table 9, the commercial oil-based coating with a higher Persoz hardness value presented with a better bonding quality compared to natural drying oils. The more complex content of commercial oil-based coatings compared to natural drying oils probably enhanced polymerization on the wood surface, which resulted in a stronger film and increased the Persoz hardness value. This result indicates that the Persoz hardness contributed to adhesion quality. A high Persoz hardness associated with good adhesion between the oil-based coating and the wood surface might contribute to an increase in hardness and prevent the coating from easily detaching or scratching.

### 3.5. Artificial Weathering

**Figure 2:**
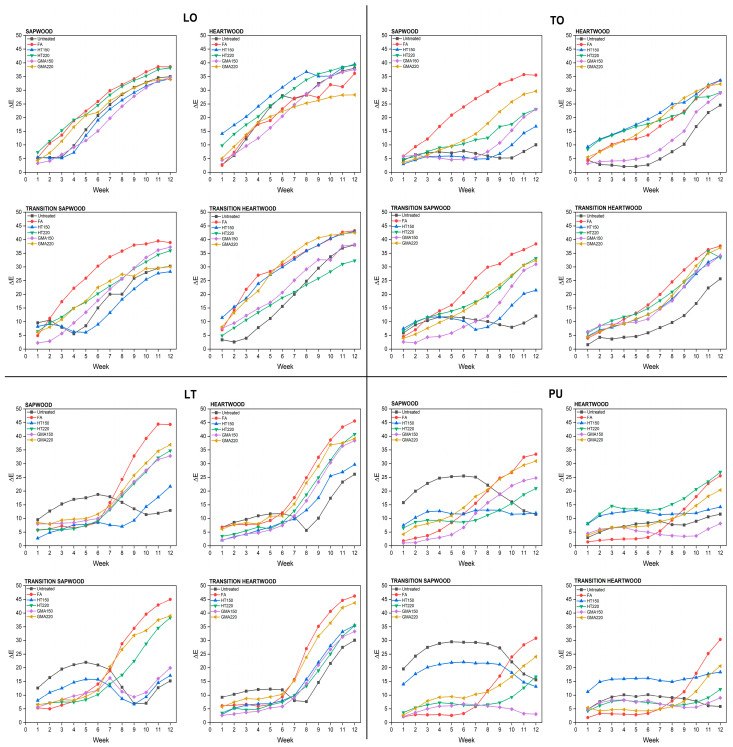
Total color changes (Δ*E*) of untreated and treated short-rotation teak wood with different oil-based coatings during artificial weathering test.

Figure 2 illustrates the total color change (Δ*E*) of untreated and treated short-rotation teak wood with various oil-based coatings during the weathering process. The total color change in short-rotation teak wood coated with oil-based materials increased as the exposure time increased. In general, the trend of total color change in heartwood is slightly lower than that in sapwood. The effect of extractives might be the main factor that provides protection against weathering factors. Furthermore, the total color change in transition sapwood and transition heartwood did not show significant differences. For LO-coated wood, the total color change increased dramatically from the initial exposure to the final exposure. TO-coated wood was slightly better in the weathering performance than LO-coated wood. The total color change in TO-coated wood increased gradually up to the 6th week and then increased dramatically after 7th week. This might be due to the higher content of unsaturated fatty acids in TO, specifically alpha-eleostearic acid, which could contribute to its UV resistance and its ability to resist light exposure without degrading as quickly as LO. Humar and Lesar [50] reported that a lower water uptake of TO indicates better water resistance compared to LO. The total color change in chemically and thermally modified teak was higher compared to that in untreated wood, especially in the FA, HT220, and GMA220 treatments. Increased hydrophobicity due to chemical and thermal modification reduced the photostability of short-rotation teak wood coated with natural drying oil. The low bonding quality of natural drying oils due to a decrease in total SFE and K-value could not provide adequate protection to chemically and thermally modified wood. The LT coating, a commercial coating material mixed from LO and TO, demonstrated good protection up to the 6th week of the weathering test. However, the total color change increased markedly after the 7th week. Interestingly, this coating was able to work successfully to protect the surface of chemically and thermally modified short-rotation teak wood. This was indicated by the lower value of total color change in chemically and thermally modified wood compared to that in untreated wood. The blend of TO and LO exhibits favorable properties, particularly in the polymerization process dominated by the conjugated fatty acid system of the tung oil fraction [47]. There was a small total color change in the PU-coated wood up to the 6th week, and it increased also after the 7th week. This coating demonstrated the lowest total discoloration. PU coatings could also protect chemically and thermally modified short-rotation teak wood against weathering factors. PU coatings contain polyurethane synthetic resins which have a more complex chemical structure and are designed to produce harder and more durable films; for example, such coatings contain UV absorber to protect against weathering factors.

**Figure 3:**
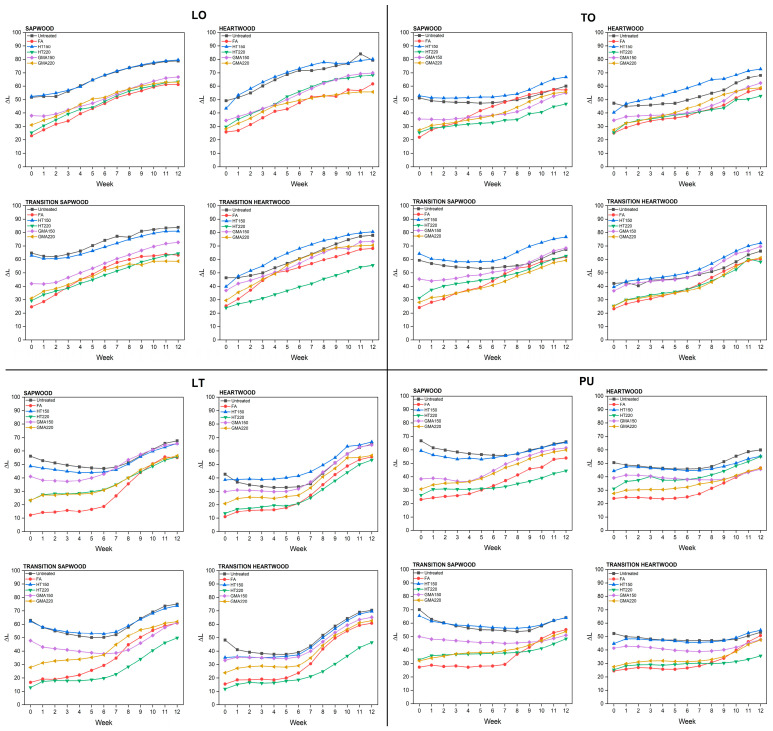
Δ*L* of untreated and treated short-rotation teak wood with different oil-based coatings during artificial weathering test.

The total color change correlated with Δ*L*, as presented in Figure 3. Dark-colored wood becomes lighter, while light-colored wood becomes darker during the weathering process [51]. A similar trend to the total color change was observed in ΔL. The wood coated with natural drying oil showed a gradual increase in Δ*L* as the exposure time increased. On the other hand, the Δ*L* change in commercial oil-based coatings was relatively stable up to 6th week and then increased in the following week. The layers of commercial coatings on chemically and thermally modified short-rotation teak wood showed a lower Δ*L* than that of the untreated. These results indicate that the improvement in photostability of chemically and thermally modified short-rotation teak wood was achieved by the application of the commercially oil-based coatings.

**Figure 4:**
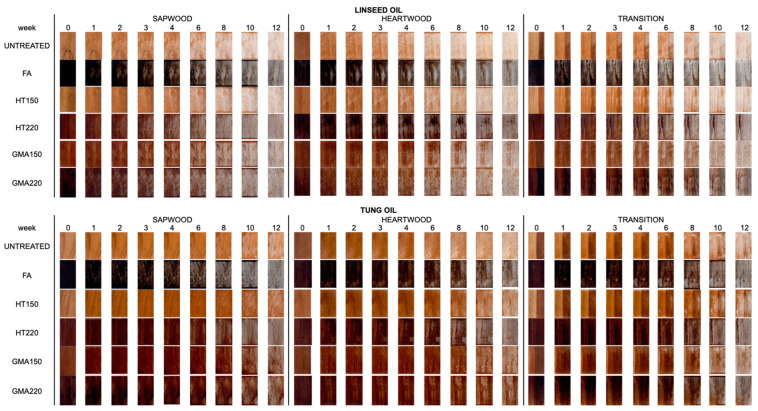

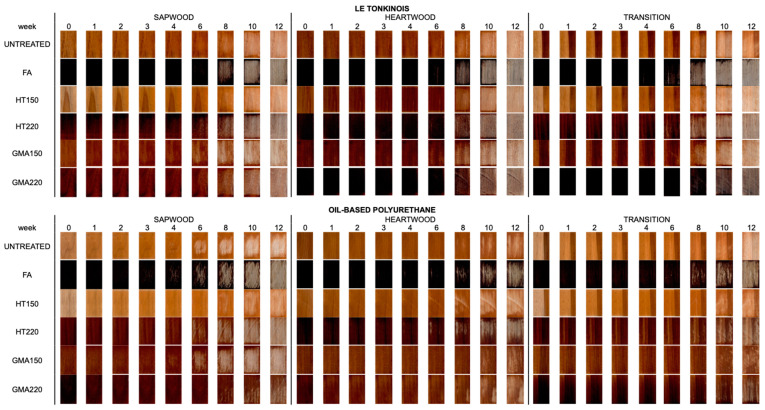
Visual color changes in untreated and treated short-rotation teak wood with linseed oil (LO), tung oil (TO), commercial oil-based coatings with a mixture of linseed oil and tung oil (LT), and commercial oil-based coatings with polyurethane resin (LB) during artificial weathering test.

The evolution of the visual color change in untreated short-rotation teak and teak treated with different oil-based coatings is shown in Figure 4. In the visual appearance of wood coated with natural drying oil, color change was observed earlier in the 3rd week for the LO coating material. Meanwhile, for the other coatings, the color change started from the 6th week. TO coatings had better characteristics for weathering protection compared to LO coatings. However, TO coatings were not able to protect the surface of chemically and thermally modified short-rotation teak. The commercial oil-based coatings could be an option to improve the weathering performance of chemically and thermally modified wood. However, commercial oil-based coatings contain volatile organic compounds (VOCs), which can be harmful to the environment and human health. A mixture of LO and TO coatings could be an environmentally friendly coating material to improve the photostability of chemically and thermally modified wood. However, a periodic maintenance process would also be required to provide optimal protection to the wood, especially for exterior applications.

## 4. Conclusions

Chemical and thermal modifications affect the surface characteristics of short-rotation teak wood. The contact angle of the reference liquids on the surface of short-rotation teak wood increases after chemical and thermal modifications, correlating to the calculation of the total SFE. The total SFE and K-value decreased after chemical and thermal treatments, leading to a decrease in the wettability of short-rotation teak. The total SFE and K-value between transition sapwood and transition heartwood were homogenized after FA and GMA220. Good bonding quality is associated with high Persoz hardness values. The TO coating shows better weathering-performance results compared to the LO coating, but the TO coating is not able to protect the modified wood surface completely. An oil-based coating with a mixture of LO and TO can be an appropriate choice to protect chemically and thermally modified wood by considering environmentally friendly reasons, like their renewable origin, allowing them to reduce their carbon footprint. The improvement in the surface protection of the chemically and thermally modified short-rotation teak by the oil-based polyurethane coatings can be considered to expand its utilization, especially for exterior applications.

## Figures and Tables

**Table 1:** The surface tension of liquid ($\gamma_{L}$), dispersive component ($\gamma_{L}^{d}$) of surface tension, and polar component of surface tension ($\gamma_{L}^{p}$).

Liquids	$\gamma_{L}$ (mN/m)	$\gamma_{L}^{d}$ (mN/m)	$\gamma_{L}^{p}$ (mN/m)
Water	72.8	21.8	51.0
Glycerol	63.4	37.0	26.4
Diiodomethane	50.8	50.8	0.00

**Table 2:** Coating properties of different oil-based coatings.

Coating Symbol	Coating Specification	Density (g/cm^3^)	Viscosity Dynamic (cst)	Solid Content (%)
LO	Linseed oil	0.926	113.5	100.0
TO	Tung oil	0.930	398.8	100.0
LO-50	50% linseed oil + 48% n-Hexane + 1% CoZi 69 +1% Ca	0.796	19.4	62.2
TO-50	50% tung oil + 48% n-Hexane + 1% CoZi 69 +1% Ca	0.799	28.8	64.8
LT	Commercial oil-based coatings with a mixture of linseed oil and tung oil provided by “Le Tonkinois”	0.900	395.7	63.6
PU	Commercial oil-based polyurethane coatings provided by “Liberon”	0.896	627.0	47.8

**Table 3:** Coating application on wood surface.

Coating	1st Layer	2nd Layer	3rd Layer
LO	LO-50	LO-50	100% LO
TO	TO-50	TO-50	100% TO
LT	95% LT + 5% white spirit	95% LT + 5% white spirit	100% LT
PU	100% PU	100% PU	100% PU

**Table 4:** Classification of cross-cut test result according to ISO 2409:2020 standard.

Classification	Description	Appearance of Surface of Cross-Cut Area from Which Flaking Has Occurred ^a^
0	The edges of the cuts are completely smooth; none of the squares of the lattice is detached.	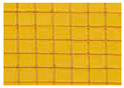
1	Detachment of small flakes of the coating at the intersections of the cuts. A cross-cut area not greater than 5% is affected.	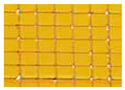
2	The coating has flaked along the edges and/or at the intersections of the cuts. A cross-cut area greater than 5%, but not greater than 15%, is affected.	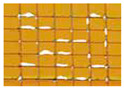
3	The coating has flaked along the edges of the cuts, partly or wholly, in large ribbons, and/or it has flaked partly or wholly on different parts of the squares. A cross-cut area greater than 15%, but not greater than 35%, is affected.	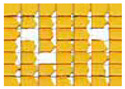
4	The coating has flaked along the edges of the cuts in large ribbons, and/or some squares have detached partly or wholly. A cross-cut area greater than 35%, but not greater than 65%, is affected.	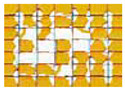
5	Any degree of flaking that cannot even be classified by classification 4.	-

^a^ The figures are examples for a cross-cut within each step of the classification. The percentages stated are based on the visual impression given by the pictures, and the same percentages will not necessarily be reproduced with digital imaging.

**Table 5:** Description of weathering cycle.

Step	Function	Temperature (°C)	Duration	Remark
1	Condensation	45	24 h	
2	Sub-cycle step 3 + 4		48 times	
3	UV	60	2.5 h	UVA-340 nm
4	Spray	37	0.5 h	6–7 L/min, nozzle size: 200 μm, UV light off

**Table 6:** Contact angle of untreated and treated wood obtained with reference liquids.

Liquids	Treatment	Contact Angle (°)			
		SW	TS	TH	HW
Glycerol	Untreated	111.85 ± 0.75 (cd)	111.32 ± 0.85 (cd)	111.96 ± 0.49 (cd)	114.03 ± 0.70 (d)
	FA	110.64 ± 0.79 (cd)	108.88 ± 1.30 (cd)	112.67 ± 1.78 (d)	113.39 ± 0.55 (d)
	HT150	110.62 ± 1.71 (cd)	109.59 ± 2.28 (cd)	111.70 ± 2.38 (cd)	112.69 ± 0.77 (d)
	HT220	109.37 ± 1.51 (cd)	112.80 ± 3.82 (d)	113.72 ± 1.18 (d)	111.74 ± 0.89 (cd)
	GMA150	107.74 ± 3.23 (bcd)	101.93 ± 4.84 (a)	113.02 ± 0.92 (d)	110.13 ± 2.81 (cd)
	GMA220	105.88 ± 1.59 (abc)	102.85 ± 11.52 (ab)	111.37 ± 1.75 (cd)	109.14 ± 3.93 (cd)
Water	Untreated	61.75 ± 3.61 (a)	78.49 ± 5.39 (b)	102.75 ± 8.14 (efg)	101.28 ± 2.66 (ef)
	FA	93.70 ± 6.60 (cd)	93.20 ± 2.66 (cd)	106.42 ± 1.67 (fg)	105.14 ± 2.52 (efg)
	HT150	91.76 ± 0.77 (cd)	91.24 ± 1.60 (c)	102.92 ± 2.79 (efg)	102.39 ± 2.00 (efg)
	HT220	106.44 ± 0.62 (fg)	103.31 ± 0.04 (efg)	106.43 ± 0.57 (fg)	109.71 ± 1.46 (g)
	GMA150	78.13 ± 5.66 (b)	89.52 ± 2.49 (c)	107.58 ± 0.90 (fg)	108.37 ± 1.07 (fg)
	GMA220	76.61 ± 4.39 (b)	80.87 ± 8.25 (b)	98.57 ± 5.19 (de)	98.57 ± 5.19 (de)
Diiodomethane	Untreated	23.02 ± 2.56 (bc)	8.67 ± 2.00 (a)	28.82 ± 0.62 (def)	31.85 ± 2.67 (fg)
	FA	36.48 ± 2.03 (h)	34.23 ± 3.37 (gh)	39.50 ± 2.46 (i)	39.59 ± 2.62 (i)
	HT150	25.90 ± 1.12 (bcd)	25.44 ± 1.85 (bcd)	21.46 ± 0.69 (b)	23.95 ± 0.67 (bc)
	HT220	30.61 ± 0.17 (efg)	25.17 ± 2.64 (bcd)	32.58 ± 2.32(fgh)	32.81 ± 2.00 (fgh)
	GMA150	24.93 ± 1.22 (bcd)	23.99 ± 0.26 (bc)	24.04 ± 0.73 (bc)	23.61 ± 0.73 (bc)
	GMA220	25.05 ± 0.78 (bcd)	23.89 ± 5.24 (bc)	31.15 ± 0.69 (def)	27.44 ± 5.15 (cde)

Note: a–i values followed by the same letters in the same sub-row (glycerol, water, and diiodomethane) do not differ significantly (α = 0.05) based on the Duncan test.

**Table 7:** Surface free energy and components of different samples.

Components	Treatment	Surface Energy (mN/m)			
		SW	TS	TH	HW
Dispersive	Untreated	44.89 ± 1.35 (ghi)	49.98 ± 0.33 (j)	43.39 ± 0.46 (defg)	41.79 ± 1.37 (cd)
	FA	39.22 ± 1.35 (ab)	40.53 ± 1.71 (bc)	37.97 ± 1.21 (a)	37.80 ± 1.50 (a)
	HT150	44.52 ± 0.43 (fghi)	44.73 ± 0.90 (fghi)	46.54 ± 0.38 (i)	45.52 ± 0.30 (hi)
	HT220	42.77 ± 0.08 (def)	44.99 ± 1.18 (ghi)	41.56 ± 1.18 (cd)	41.67 ± 0.91 (cd)
	GMA150	44.71 ± 0.45 (fghi)	45.61 ± 0.13 (hi)	45.57 ± 0.33 (gh)	45.85 ± 0.36 (hi)
	GMA220	44.68 ± 0.39 (fghi)	45.17 ± 2.38 (ghi)	42.25 ± 0.51 (cde)	43.98 ± 2.10 (efgh)
Polar	Untreated	0.23± 0.33 (ab)	1.16 ± 0.63 (abcd)	3.60 ± 1.26 (ghi)	3.22 ± 0.52 (gh)
	FA	1.36 ± 0.95 (bcd)	1.17 ± 0.17 (abcd)	2.91 ± 0.57 (fg)	2.83 ± 0.71 (fg)
	HT150	1.92 ± 0.26 (def)	1.73 ± 0.23 (cde)	4.36 ± 0.64 (ijk)	4.21 ± 0.20 (hijk)
	HT220	3.47 ± 0.19 (ghi)	4.30 ± 0.90 (hijk)	4.04 ± 0.40 (hij)	4.18 ± 0.26 (hijk)
	GMA150	0.36 ± 0.37 (ab)	0.82 ± 0.53 (abcd)	5.22 ± 0.15 (k)	4.78 ± 0.42 (jk)
	GMA220	0.17 ± 0.29 (a)	0.76 ± 0.92 (abc)	2.94 ± 0.66 (fg)	2.57 ± 0.97 (efg)
SFE total	Untreated	45.13 ± 1.06 (b)	51.13 ± 0.44 (c)	46.99 ± 1.65 (b)	45.00 ± 1.17 (b)
	FA	40.58 ± 2.28 (a)	41.70 ± 1.67 (a)	40.88 ± 1.75 (a)	40.58 ± 2.28 (a)
	HT150	46.44 ± 0.68 (b)	49.29 ± 1.61 (c)	50.90 ± 0.52 (c)	49.72 ± 0.44 (c)
	HT220	46.24 ± 0.15 (b)	46.46 ± 0.68 (b)	45.61 ± 1.43 (b)	45.86 ± 1.14 (b)
	GMA150	45.07 ± 0.17 (b)	46.40 ± 0.53 (b)	50.79 ± 0.29 (c)	50.66 ± 0.46 (c)
	GMA220	44.85 ± 0.61 (b)	45.92 ± 2.01 (b)	45.19 ± 1.05 (b)	46.55 ± 2.28 (b)

Note: a–k values followed by the same letters in the same sub-row (dispersive, polar and SFE total) do not differ significantly (α = 0.05) based on the Duncan test.

**Table 8:** K-value of oil-based coatings on different wood parts for untreated and treated of short-rotation teak wood.

Coatings	Treatment	K-Value			
		SW	TS	TH	HW
LO	Untreated	0.54 ± 0.04 (j)	0.51 ± 0.05 (ij)	0.36 ± 0.02 (def)	0.39 ± 0.02 (fg)
	FA	0.24 ± 0.02 (a)	0.24 ± 0.04 (ab)	0.25 ± 0.03 (ab)	0.25 ± 0.03 (abc)
	HT150	0.44 ± 0.03 (ghi)	0.49 ± 0.06 (hij)	0.43 ± 0.02 (gh)	0.25 ± 0.02 (abc)
	HT220	0.29 ± 0.05 (abcd)	0.31 ± 0.03 (abcde)	0.31 ± 0.02 (abcd)	0.25 ± 0.11 (ab)
	GMA150	0.38 ± 0.03 (efg)	0.44 ± 0.02 (gh)	0.43 ± 0.04 (gh)	0.38 ± 0.02 (efg)
	GMA220	0.31 ± 0.03 (bcdef)	0.34 ± 0.01 (cdef)	0.33 ± 0.03 (cdef)	0.29 ± 0.06 (abcd)
TO	Untreated	0.51 ± 0.06 (i)	0.51 ± 0.01 (i)	0.32 ± 0.06 (bcde)	0.38 ± 0.03 (ef)
	FA	0.37 ± 0.02 (def)	0.34 ± 0.04 (bcde)	0.33 ± 0.01 (bcde)	0.35 ± 0.03 (bcdef)
	HT150	0.45 ± 0.05 (gh)	0.44 ± 0.02 (gh)	0.34 ± 0.02 (bcde)	0.31 ± 0.05 (abcd)
	HT220	0.35 ± 0.01 (bcde)	0.41 ± 0.03 (fg)	0.30 ± 0.06 (abc)	0.25 ± 0.02 (a)
	GMA150	0.49 ± 0.02 (hi)	0.32 ± 0.04 (bcde)	0.35 ± 0.02 (bcde)	0.37 ± 0.01 (def)
	GMA220	0.36 ± 0.03 (cdef)	0.29 ± 0.04 (ab)	0.31 ± 0.02 (abcd)	0.32 ± 0.02 (bcde)
LT	Untreated	0.35 ± 0.04 (abcdef)	0.39 ± 0.06 (def)	0.34 ± 0.03 (abcdef)	0.30 ± 0.05 (abc)
	FA	0.29 ± 0.02 (abc)	0.29 ± 0.05 (abc)	0.27 ± 0.03 (a)	0.27 ± 0.02 (a)
	HT150	0.34 ± 0.01 (abcde)	0.42 ± 0.07 (f)	0.34 ± 0.01 (abcdef)	0.34 ± 0.04 (abcdef)
	HT220	0.33 ± 0.04 (abcde)	0.39 ± 0.05 (ef)	0.31 ± 0.05 (abcdef)	0.32 ± 0.03 (abcde)
	GMA150	0.36 ± 0.06 (bcdef)	0.36 ± 0.07 (cdef)	0.34 ± 0.02 (abcd)	0.32 ± 0.00 (abcde)
	GMA220	0.34 ± 0.05 (abcde)	0.36 ± 0.04 (bcdef)	0.35 ± 0.05 (bcdef)	0.28 ± 0.03 (ab)
PU	Untreated	0.34 ± 0.03 (f)	0.36 ± 0.03 (f)	0.34 ± 0.04 (f)	0.21 ± 0.09 (a)
	FA	0.28 ± 0.04 (abcdef)	0.24 ± 0.05 (abcde)	0.25 ± 0.02 (abcde)	0.25 ± 0.04 (bcdef)
	HT150	0.30 ± 0.03 (bcdef)	0.34 ± 0.04 (f)	0.31 ± 0.03 (def)	0.30 ± 0.09 (bcdef)
	HT220	0.24 ± 0.02 (abcd)	0.22 ± 0.05 (ab)	0.21 ± 0.02 (ab)	0.22 ± 0.04 (ab)
	GMA150	0.34 ± 0.03 (ab)	0.30 ± 0.03 (bcdef)	0.25 ± 0.00 (abcd)	0.32 ± 0.01 (abcde)
	GMA220	0.30 ± 0.05 (cdef)	0.28 ± 0.02 (abcdef)	0.25 ± 0.04 (abcde)	0.20 ± 0.08 (a)

Note: a–j values followed by the same letters in the same sub-row (LO, TO, LT, and PU) do not differ significantly (α = 0.05) based on the Duncan test.

**Table 9:** Bonding quality of different oil-based coating on untreated and treated short-rotation teak wood.

Coating	Treatments	Bonding Quality		
		SW	TW	HW
LO	Untreated	0	0	0
	FA	2	1	2
	HT150	0	0	1
	HT220	1	1	1
	GMA150	1	1	1
	GMA220	1	1	1
TO	Untreated	0	0	0
	FA	1	1	1
	HT150	0	0	0
	HT220	1	0	1
	GMA150	0	0	1
	GMA220	0	0	1
LT	Untreated	0	0	0
	FA	0	0	0
	HT150	0	0	0
	HT220	0	0	0
	GMA150	0	0	0
	GMA220	0	0	0
LB	Untreated	0	0	0
	FA	0	0	0
	HT150	0	0	0
	HT220	0	0	0
	GMA150	0	0	0
	GMA220	0	0	0

## Data Availability

Data are contained and presented within the article.
